# Ultrasound estimates of muscle quality in older adults: reliability and comparison of Photoshop and ImageJ for the grayscale analysis of muscle echogenicity

**DOI:** 10.7717/peerj.1721

**Published:** 2016-02-22

**Authors:** Michael O. Harris-Love, Bryant A. Seamon, Carla Teixeira, Catheeja Ismail

**Affiliations:** 1 Muscle Morphology, Mechanics and Performance Laboratory, Clinical Research Center, Washington DC Veterans Affairs Medical Center, Washington, DC, United States; 2 Department of Exercise and Nutrition Sciences, Milken Institute School of Public Health, George Washington University, Washington, DC, United States; 3 Geriatrics and Extended Care Service, Washington DC Veterans Affairs Medical Center, Washington, DC, United States; 4 Physical Medicine & Rehabilitation Service, Washington DC Veterans Affairs Medical Center, Washington, DC, United States; 5 The School of Kinesiology and Health Studies, Queen’s University, Kingston, Ontario, Canada

**Keywords:** Ultrasound, Grayscale histogram analysis, Muscle echogenicity, ImageJ, Photoshop, Geriatrics

## Abstract

**Background.** Quantitative diagnostic ultrasound imaging has been proposed as a method of estimating muscle quality using measures of echogenicity. The Rectangular Marquee Tool (RMT) and the Free Hand Tool (FHT) are two types of editing features used in Photoshop and ImageJ for determining a region of interest (ROI) within an ultrasound image. The primary objective of this study is to determine the intrarater and interrater reliability of Photoshop and ImageJ for the estimate of muscle tissue echogenicity in older adults via grayscale histogram analysis. The secondary objective is to compare the mean grayscale values obtained using both the RMT and FHT methods across both image analysis platforms.

**Methods.** This cross-sectional observational study features 18 community-dwelling men (age = 61.5 ± 2.32 years). Longitudinal views of the rectus femoris were captured using B-mode ultrasound. The ROI for each scan was selected by 2 examiners using the RMT and FHT methods from each software program. Their reliability is assessed using intraclass correlation coefficients (ICCs) and the standard error of the measurement (SEM). Measurement agreement for these values is depicted using Bland-Altman plots. A paired *t*-test is used to determine mean differences in echogenicity expressed as grayscale values using the RMT and FHT methods to select the post-image acquisition ROI. The degree of association among ROI selection methods and image analysis platforms is analyzed using the coefficient of determination (*R*^2^).

**Results.** The raters demonstrated excellent intrarater and interrater reliability using the RMT and FHT methods across both platforms (lower bound 95% CI ICC = .97–.99, *p* < .001). Mean differences between the echogenicity estimates obtained with the RMT and FHT methods was .87 grayscale levels (95% CI [.54–1.21], *p* < .0001) using data obtained with both programs. The SEM for Photoshop was .97 and 1.05 grayscale levels when using the RMT and FHT ROI selection methods, respectively. Comparatively, the SEM values were .72 and .81 grayscale levels, respectively, when using the RMT and FHT ROI selection methods in ImageJ. Uniform coefficients of determination (*R*^2^ = .96–.99, *p* < .001) indicate strong positive associations among the grayscale histogram analysis measurement conditions independent of the ROI selection methods and imaging platform.

**Conclusion.** Our method for evaluating muscle echogenicity demonstrated a high degree of intrarater and interrater reliability using both the RMT and FHT methods across 2 common image analysis platforms. The minimal measurement error exhibited by the examiners demonstrates that the ROI selection methods used with Photoshop and ImageJ are suitable for the post-acquisition image analysis of tissue echogenicity in older adults.

The rapid expansion of point-of-care sonography in hospital settings reflects the significant evolution of diagnostic ultrasound (Cardenas-Garcia & Mayo, 2015). This imaging modality has been used clinically for disparate purposes such as aiding needle placement for nerve blocks and aspiration procedures (Klaastad, Sauter & Dodgson, 2009; Fung et al., 2014), and providing biofeedback of the trunk musculature during physical rehabilitation (Giggins, Persson & Caulfield, 2013). In addition, the research applications of sonography within the field of exercise physiology and other movement science disciplines have greatly impacted the use of quantitative musculoskeletal ultrasound to characterize muscle architecture (Reeves et al., 2009). Investigators have used sonography to measure post-exercise changes in muscle size, determine pennation angle, measure muscle fascicle length, and estimate cross-sectional area (Kwah et al., 2013; Franchi et al., 2014). Quantitative musculoskeletal ultrasound has also been proposed as an alternative imaging modality to provide estimates of muscle quality based on tissue composition (Sipilä & Suominen, 1993). Diminished muscle composition due to adipose tissue infiltration or increased fibrotic tissue is associated with impaired muscle mechanics that may contribute to functional deficits (Sipilä & Suominen, 1994; Maly et al., 2013; Rahemi, Nigam & Wakeling, 2015). While intramuscular adipose tissue can be assessed using computed tomography (CT) imaging (Lang et al., 2010), the serial use of the modality for this purpose is precluded due to exposure to ionizing radiation. Magnetic resonance imaging (MRI) has also been used to estimate intramuscular adipose tissue (Wroblewski et al., 2011). However, the wide utilization of MRI for the assessment of age-related declines in muscle quality is not feasible due to cost constraints and general access issues. Echogenicity, the characteristic of tissue or other material to reflect ultrasound waves, is typically used as a sonographic estimate of muscle quality. Measures of muscle echogenicity have been shown to be significantly associated intramuscular adipose tissue, rather than fibrotic tissue, based on the biochemical analysis of biopsied tissue samples from 82 people with muscle pathology (Reimers et al., 1993). Lower estimates of muscle echogenicity have also been shown to be associated with measures of high muscle density in older women (Sipilä & Suominen, 1993). Consequently, the assessment of muscle quality via echogenicity measures has been used as a means to discriminate among individuals with and without muscle abnormalities (Whittaker et al., 2007; Pillen & Van Alfen, 2011). Echogenicity measures have demonstrated value in the assessment of neuromuscular diseases, and may be an important factor in observed muscle performance deficits in older adults (Zaidman et al., 2010; Fukumoto et al., 2012; Ismail et al., 2015).

While echogenicity values can be derived from the digital backscattered radio-frequency (RF) signal, this approach is rarely viable in clinical environments due to the limitations of many commercial ultrasound machines and the need for custom signal processing (Zaidman, Holland & Hughes, 2012). Consequently, the quantitative estimation of echogenicity in clinical settings is often through grayscale histogram analysis. This imaging analysis technique involves the construction of a plot featuring the number of pixels associated with a given region of interest (ROI) within intervals determined by intensity level (Pillen et al., 2009; Iftekharuddin & Awwal, 2012). Post-image acquisition analysis may be performed using a variety of image editing programs. A commonly cited program for grayscale histogram analysis is Photoshop (Adobe Systems, San Jose, CA), which has been broadly used for clinical applications ranging from the quantitative analysis of endothelial damage to the measurement of skeletal muscle echogenicity in older adults (Saad et al., 2008; Watanabe et al., 2013; Casella et al., 2015). A widely used alternative to the commercially available Photoshop program is ImageJ, a public-domain Java-based image processing and analysis program developed by Wayne Rasband of the National Institute of Mental Health at NIH (Rasband, 1997; Schneider, Rasband & Eliceiri, 2012). ImageJ has been extensively used for image processing in immunohistochemistry (Schneider, Rasband & Eliceiri, 2012), tissue segmentation in microscopy images (Collins, 2007), and muscle morphometry measurements (Fortin & Battié, 2012). Photoshop and ImageJ have both been cited as being among the most frequently used image processing and analysis programs (Nanes, 2015). Moreover, the origin of ImageJ within a Federal biomedical institution allows for the download of the program through the Veterans Health Administration network security system, thus facilitating its use within Veterans Affairs medical centers. Many image analysis platforms are available and vary based on file type constraints, software customization and flexibility, hardware requirements, cost limitations, and image visualization needs (e.g., confocal microscopy, CT imaging, sonography, etc.). Options range from commercially available software such as Analyze (AnalyzeDirect, Inc., Kansas, USA) and sliceOmatic (TomoVision, Canada), to open source software options such as OpenCV, GNU Image Manipulation Program, the Medical Imaging Interaction Toolkit, MIPAV (Medical Image Processing, Analysis, and Visualization), and OsiriX. Photoshop and ImageJ are featured in this report given their availability at our Federal hospital and because the software capabilities are appropriate for the grayscale histogram analysis required to estimate echogenicity in sonographic images. In addition, the selected image analysis platforms allow for a comparison between a widely used commercial software program and an established open source option.

Both Photoshop and ImageJ have a variety of selection tools for determining an ROI within an image. Two commonly used methods include the semi-automated creation of a square or rectangular ROI region, or tracing the ROI region using a series of line segments that closely align with the targeted anatomical or morphological structure. In this work, the “Rectangular Marquee Tool” (RMT) is the term used to describe the Rectangular Marquee selection method in Photoshop and the Rectangular selection method in ImageJ. Similarly, the “Free Hand Tool” (FHT) is the term used to describe the Magnetic Lasso Tool (which requires manual adjustments to the semi-automated ROI selections) in Photoshop and the fully manual Freehand selection method in ImageJ. Both ROI selection methods have relative advantages related to the examiner time used to determine the tissue boundaries included in the analysis (RMT), and the accuracy of the selected ROI based on the fascial borders of the targeted muscle (FHT). There is a need to determine if direct comparisons can be made between software programs for the ROI selection used to obtain mean grayscale values. Moreover, it is uncertain if meaningful intrarater or interrater differences exist in the measurement of mean grayscale values based on the post-image acquisition ROI selection method (i.e., RMT versus FHT). Therefore, the primary objective of this study is to determine the intrarater and interrater reliability of Photoshop and ImageJ for the estimate of muscle tissue echogenicity in older adults via grayscale histogram analysis. The secondary objective was to compare the mean grayscale values obtained using both the RMT versus FHT across both image analysis programs.

## Materials and Methods

### Study design and setting

This cross-sectional observational study was conducted to determine the reliability and agreement of echogenicity measures at the rectus femoris of older male veterans at a VA Medical Center. Image acquisition was completed in a clinical environment within an outpatient unit of a Physical Medicine and Rehabilitation department. Post-image acquisition analysis was undertaken within a laboratory at the medical center.

### Participants

Eighteen community-dwelling older adults were enrolled for participation in the study at the Washington DC Veterans Affairs Medical Center (DC VAMC). All of the participants were generally healthy, community-dwelling older male veterans (*n* = 18, age = 61.5 ± 2.32 years; BMI: 27.6 ± 1.15). The study was approved by the DC VAMC Research and Development Institutional Review Board (IRB; #01671), and registered with Clinicaltrials.gov (NCT02277236). Signed informed consent was obtained from all study participants prior to data collection. The novice examiners involved in the post-image acquisition analysis were two research associates in a laboratory associated with a Physical Medicine and Rehabilitation department with active protocols involving quantitative ultrasound. The background of these examiners included physical therapy (B.A.S.) and exercise science (C.T.). The examiners were trained over a three-month period by a clinical sonographer and a rehabilitation scientist (both with over ten years of sonography experience) regarding ultrasound image capture, sonographic artifacts, and software-aided image analysis. Deidentified sample images were analyzed with an instructor using both software programs prior to data collection.

**Figure 1:**
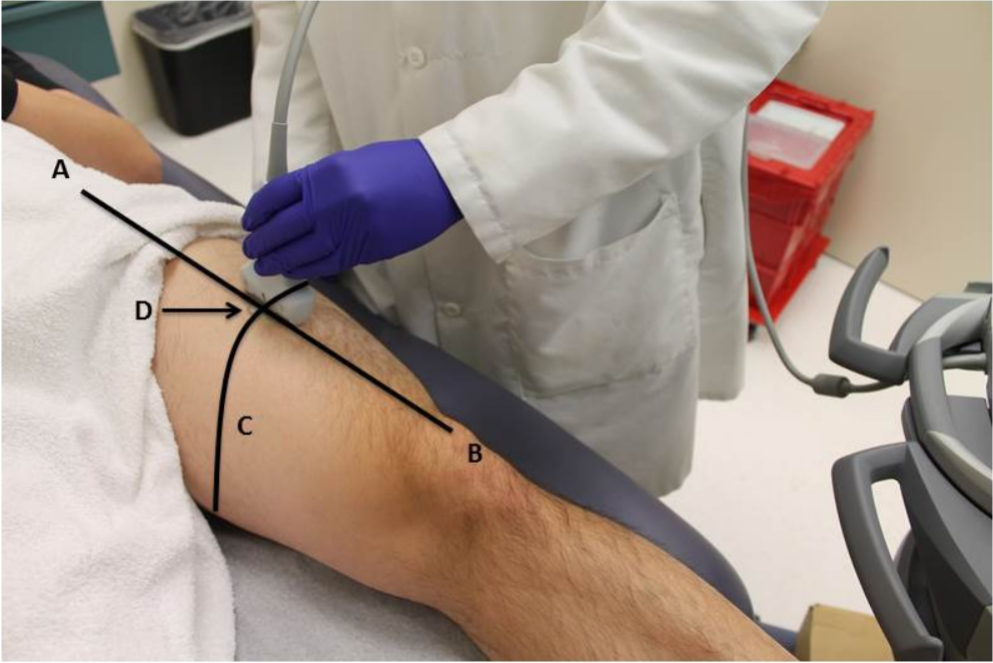
Participant positioning during ultrasound scanning procedure. The scanning site on the anterior surface of the thigh (dominant side) was located by obtaining a measurement in cm from the anterior superior iliac spine (A) and the superior pole of the patella (B). This measurement distance was bisected (C) and the midpoint of line was marked with an indelible marker. The participant was then positioned on a plinth in supine with the leg fully supported. A linear ultrasound transducer was placed at the marked scanning site (D) and the transducer was positioned to capture a longitudinal view of the rectus femoris as depicted in the figure. A sufficient amount of water-soluble transmission gel was used during scanning for optimal acoustic contact with the imaging site, and minimal pressure was applied to the sound transducer in order to limit tissue deformation.

### Procedures

Quantitative ultrasound scanning and image capture were completed using a diagnostic sonography machine (SonoSite M-Turbo 1.1.2; SonoSite, Inc., Bothell, WA, USA) with a 13.6 MHz linear array transducer and B-mode scanning. The ultrasound machine was operated using its default gain levels and compression was governed by the factory presets (i.e., the “resolution” setting in the “musculoskeletal” scanning preset). The scanning site on the anterior surface of the thigh (dominant side) was located by bisecting the distance in cm between the anterior superior iliac spine and the superior pole of the patella, and then marked with an indelible marker (Fig. 1). This approach facilitated the consistent image capture of the rectus femoris within the field of view and has been used in previous studies (Ismail et al., 2015; Hernandez et al., 2015). Longitudinal view image capture was completed with the participant lying supine on a plinth and positioning the ultrasound transducer to be oriented 90° to the muscle bundles. A sufficient amount of water-soluble transmission gel was used during scanning for optimal acoustic contact with the imaging site, and minimal pressure was applied to the transducer in order to limit tissue deformation. Scanning was completed by a separate examiner from the laboratory with at least one year of quantitative ultrasound experience. The examiner responsible for scanning is part of a laboratory that has demonstrated strong interrater measurement consistency, exhibiting a coefficient of variation of ≤2.9% for morphometry using a calibration phantom, and high interrater reliability (ICC_2,*k*_ = .992, *p* < .001) for the assessment of echogenicity at the rectus femoris via grayscale histogram analysis (Harris-Love et al., 2015).

**Figure 2:**
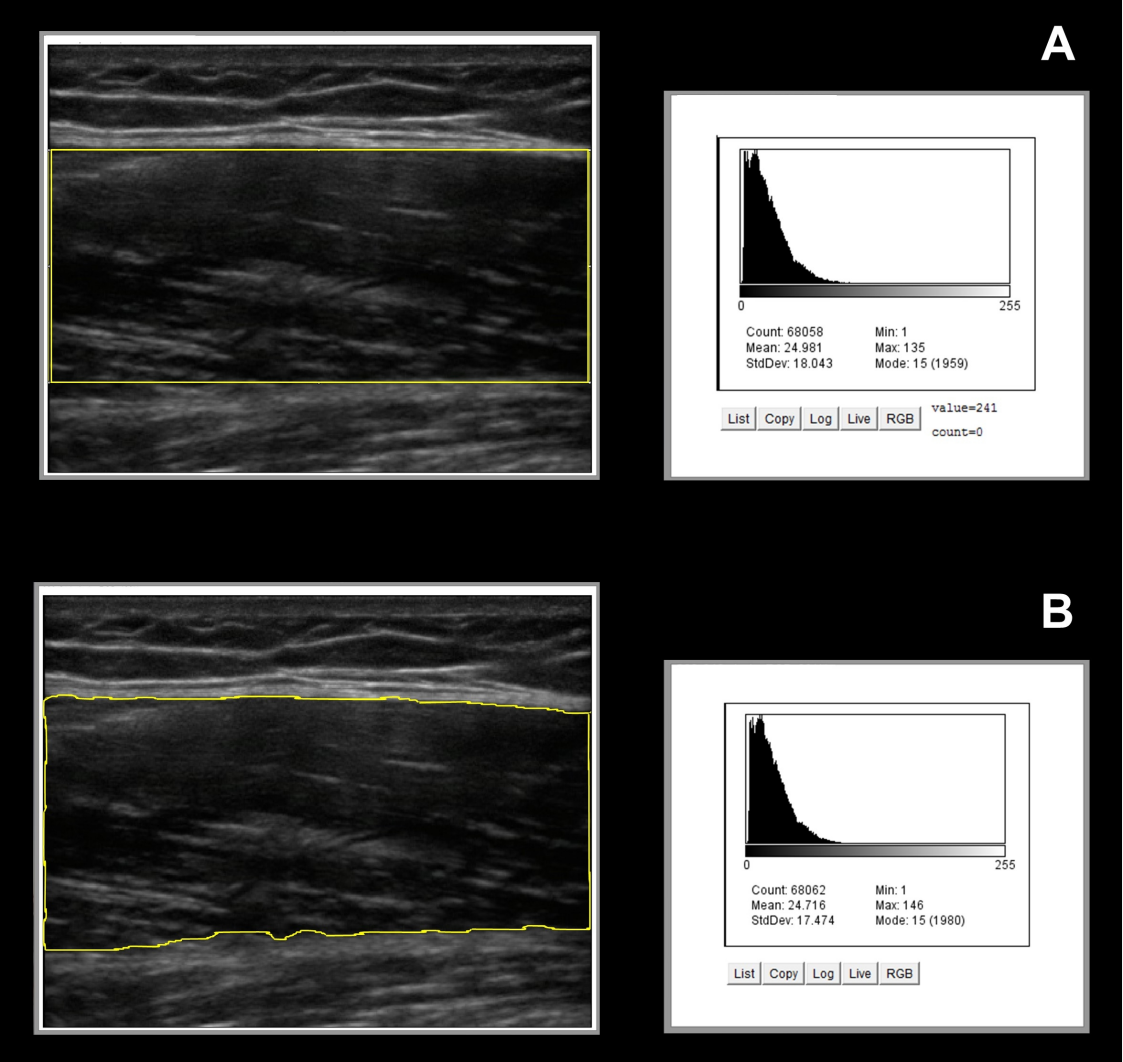
Image analysis ROI selections and the corresponding grayscale histogram values. The exemplar longitudinal images of the rectus femoris feature an overlay graphic of the ROI selection using the Rectangular Marquee Tool (A) and Free Hand Tool (B) provided by the ImageJ software. The corresponding grayscale histogram analysis data for each sonographic image shows similar estimates of rectus femoris echogenicity using both ROI selection methods. (ROI, region of interest. Post-analysis image enhancement used only to emphasize the ROI selection borders in the figure.)

The two examiners responsible for post-image acquisition analysis independently measured the echogenicity of the rectus femoris in the longitudinal view. Mean grayscale values were obtained for each scan using two image editing programs: Photoshop (version 6.0) and ImageJ (version 1.48). Each examiner selected the ROI for each scan using the Rectangular Marquee Tool (RMT) and the Free Hand Tool (FHT). The measurement procedure included obtaining the ROI within the superior and inferior fascial borders of the muscle and the lateral borders of the muscle defined by the field of view, as adapted from Pillen and associates (2003). In select instances when a portion of a fascial border was poorly visualized, the examiner used the trajectory of the visible fascial border to complete the ROI selection. Additionally, the geometric configuration of the RMT ROI was not expected to perfectly conform to the fascial planes of the rectus femoris. Therefore, the examiners were instructed to maximize the congruency between the selected ROI using the RMT and the superior and inferior fascial borders of the muscle (Fig. 2). All intrasession grayscale measures were completed twice and the mean value was used for the data analysis. The assessment order for each image was separately randomized for each examiner to minimize measurement bias (VassarStats random number generator) (Lowry, 2004), and the assessment sessions were separated by one week to minimize the effects of recall on the selection of the ROI. Masking was employed regarding the initial measurement values, and no information concerning the results of the grayscale histogram analysis was shared between the examiners during the data collection period.

### Data analysis

All data and variance distributions are normal based on the Shapiro–Wilk and Levene’s tests, and conveyed as means and standard deviations. Echogenicity levels are expressed as grayscale values (0–255, unitless values). The relative reliability of the examiners for the grayscale histogram analysis is estimated using intraclass correlation coefficients (ICC). The ICC_2,__*k*_ is used to determine the interrater reliability using a 2-way mixed model absolute agreement approach, and the ICC_3,__*k*_ is used to determine the intrarater reliability using a 2-way random model consistency approach (Portney & Watkins, 2009). Portney & Watkins (2009) report that clinical measurements should attain a coefficient of ≥.75 for adequate reliability, although some assessments may need to exceed this standard depending on the application. Munro (2001) has stated that statistical estimates based on non-random sampling may compromise inferences from a given data set. Therefore, we will examine the lower bound point estimate of the reliability coefficients featured in this report (i.e., 95% CI of the ICCs) to account for the inherent uncertainty associated with samples of convenience (Eliasziw et al., 1994). The Portney & Watkins (2009) criteria is used to interpret the ICC findings: 00–.49 = poor reliability, .50–.74 = moderate reliability, and .75–1.00 = excellent reliability. The standard error of the measurement (SEM) is calculated to provide an estimate of absolute reliability of the examiners’ grayscale histogram analysis (within the given scale of measurement). Measurement agreement for these values is depicted visually using the 95% limits of agreement method as suggested by Bland and Altman (Giavarina, 2015). The paired *t*-test is used to determine mean differences in echogenicity expressed as grayscale values using the RMT and FHT methods to select the post-image acquisition ROI. The degree of association among ROI selection methods and image analysis platforms is displayed visually as a scatter plot and analyzed using the coefficient of determination (*R*^2^). Statistical analyses were performed using PASW Statistics for Windows, Version 18.0 (SPSS Inc., Chicago, IL, USA). The *α* level was set at .05, and two-tailed *p* values < .05 were considered significant for all inferential statistics.

## Results

### Grayscale histogram analysis reliability and measurement error

The examiners demonstrated excellent reliability using the RMT and FHT methods to select the ROI across both image analysis platforms. The intraclass correlation coefficients (ICCs) ranged from .97 to .99 (*p* < .001; lower bound 95% CI) for both intrarater and interrater measurement performance. The aggregate measures for both examiners reveal that mean grayscale values differed by 1.6% for Photoshop in comparison to ImageJ when using the RMT method to select the ROI. The mean grayscale values differed by 2.5% across these image analysis platforms when using the FHT method to select the ROI. Measurement error for both ROI selection methods was nominal when using either Photoshop or ImageJ for grayscale histogram analysis. The SEM for Photoshop was .97 and 1.05 grayscale levels when using the RMT and FHT ROI selection methods, respectively. Comparatively, the SEM values were .72 and .81 grayscale levels, respectively, when using the RMT and FHT ROI selection methods in ImageJ. The data regarding grayscale histogram analysis reliability and measurement error is presented in Table 1.

**Table 1:** Intrarater and interrater reliability for grayscale histogram analysis. Relative reliability of the ROI selection method for grayscale histogram analysis is determined using intraclass correlation coefficients (ICC), and absolute reliability is expressed via the standard error of measurement (SEM). Both the Rectangular Marquee Tool and Free Hand Tool displayed comparable levels of reliability across image processing platforms and examiners, with the echogenicity estimates obtained with ImageJ software exhibiting slightly lower SEM values. Measurement values are in grayscale levels.

		Photoshop			ImageJ		
		$\bar {X}\pm SD$	ICC (95% CI)^a^	SEM	$\bar {X}\pm SD$	ICC (95% CI)^a^	SEM
*Examiner 1*^b^	Rectangular Marquee Tool	28.09 ± 11.51	.993 (.982)	.96	27.72 ± 11.45	.996 (.990)	.72
	Free Hand Tool	27.52 ± 11.77	.992 (.978)	1.05	26.55 ± 11.38	.995 (.986)	.80
*Examiner 2*^b^	Rectangular Marquee Tool	28.19 ± 11.64	.993 (.982)	.97	27.69 ± 11.39	.996 (.990)	.72
	Free Hand Tool	27.35 ± 11.68	.992 (.978)	1.04	27.01 ± 11.63	.995 (.986)	.82
*Both examiners*^c^	Rectangular Marquee Tool	28.15 ± 11.57	.993 (.982)	.97	27.71 ± 11.42	.996 (.990)	.72
	Free Hand Tool	27.44 ± 11.72	.992 (.978)	1.05	26.78 ± 11.51	.995 (.986)	.81

**Notes.**

a one-sided lower limit (CI = confidence interval).

b ICC_3,*k*_ used to determine intrarater reliability.

c ICC_2,*k*_ used to determine interrater reliability.

*p* < .001 for all ICC values.

(SEM, standard error of the measurement).

### Examiner agreement based on ROI selection method and image analysis platform

**Figure 3:**
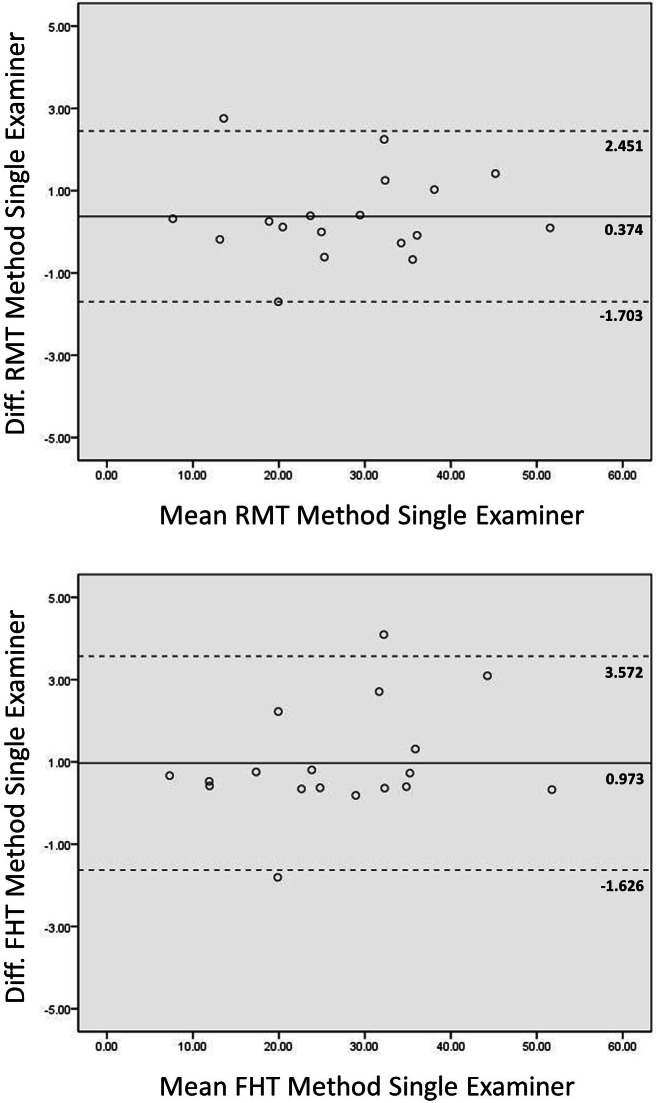
Intrarater Bland-Altman plots across image processing platforms. Bland-Altman plots for agreement between grayscale analysis measures for the rectus femoris muscle using Photoshop and ImageJ are shown. The mean of two measures obtained from both image processing platforms, as assessed by Examiner 1, is depicted on the *x*-axis. The difference between the means of these measures are depicted on the *y*-axis. The mean difference and limits of agreement (1.96 ⋅ standard deviation) are represented by the horizontal lines parallel to the *x*-axis. (RMT, Rectangular Marquee Tool; FHT, Free Hand Tool; diff., difference; grayscale level range = 0–255.)

The Bland-Altman plots in Fig. 3 depict the agreement between the grayscale measurements across the two image analysis platforms for Examiner 1. The limits of agreement for grayscale measures obtained using the FHT method for ROI selection were wider than comparable measures obtained using the RMT method. The mean difference between the echogenicity estimates obtained with the RMT and FHT methods was .87 grayscale levels (95% CI [.54–1.21]; *p* < .0001) using data obtained with both Photoshop and ImageJ.

**Figure 4:**
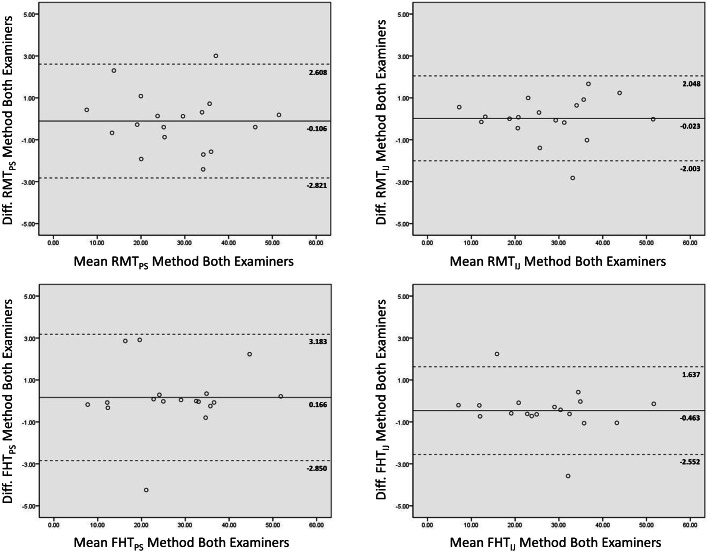
Interrater Bland-Altman plots for two examiners. The Bland-Altman plots depict the agreement between two novice examiners obtaining the grayscale analysis measures for the rectus femoris muscle. Plots are provided showing the observed measurement agreement using both of the image processing platforms, and ROI selection methods. The mean of the measures obtained from both examiners is depicted on the *x*-axis. The difference between the means of these measures are depicted on the *y*-axis. The mean difference and limits of agreement (1.96 ⋅ standard deviation) are represented by the horizontal lines parallel to the *x*-axis. (ROI, region of interest; PS, Photoshop; IJ, ImageJ; RMT, Rectangular Marquee Tool; FHT, Free Hand Tool; diff., difference; grayscale level range = 0–255.)

**Figure 5:**
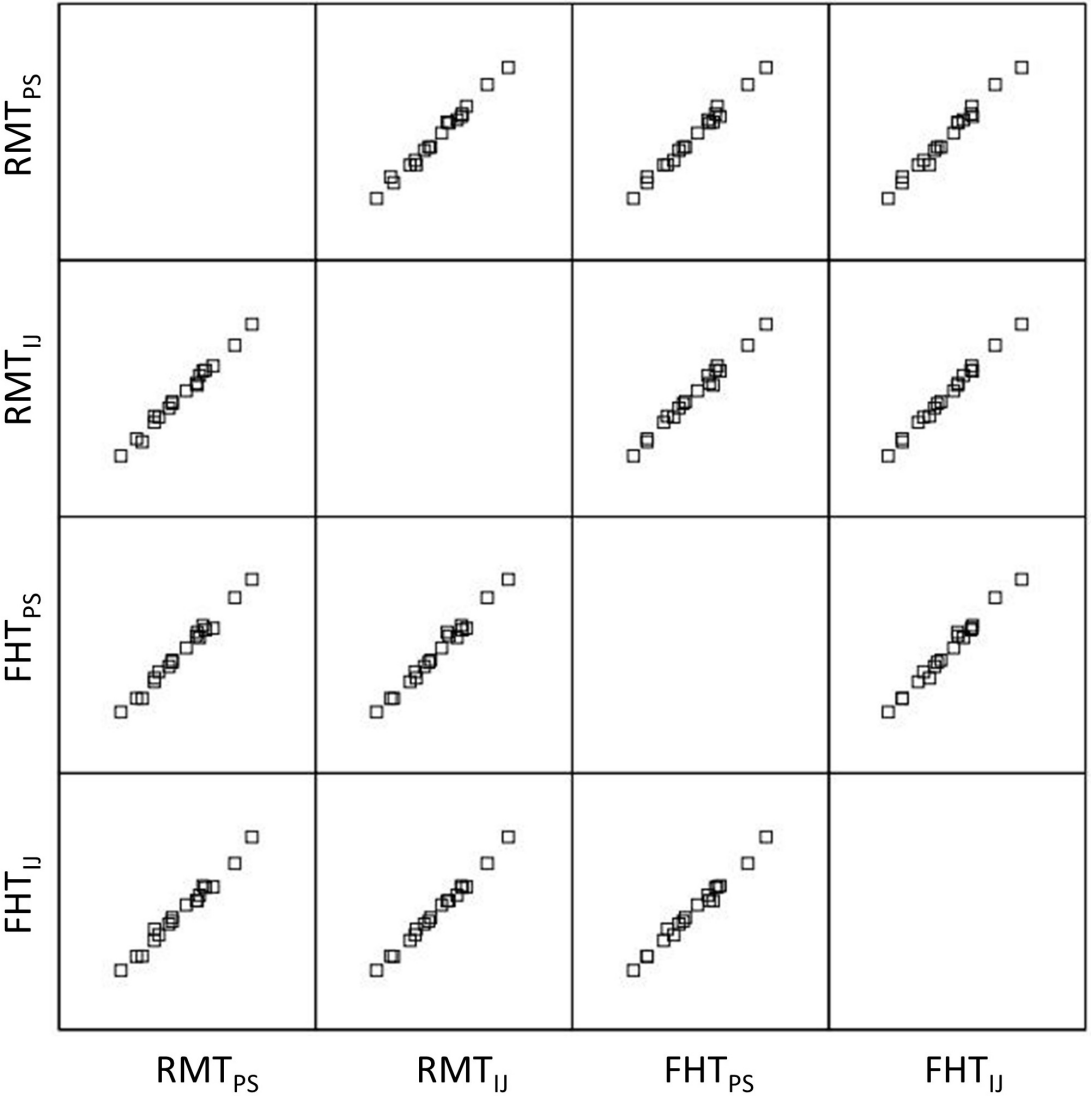
Matrix scatter plots across both image processing platforms and ROI selection methods. The matrix scatter plots show the relationships among the grayscale values obtained by Examiner 1 using both of the image processing platforms and ROI selection methods. The coefficient of determination (*R*^2^) values for all linear regression analyses within the matrix were .96–.99 (*p* < .001). Specific matrix combinations such as RMT_*PS*_ × RMT_*IJ*_ and FHT_*PS*_ × FHT_*IJ*_ suggest that similar ROI selection methods were highly associated across imaging platforms. Also, matrix combinations such as RMT_*PS*_ x FHT_*PS*_ and RMT_*IJ*_ × FHT_*IJ*_ indicate that differing ROI selection methods using Photoshop or ImageJ yield significant positive relationships. (ROI, region of interest; PS, Photoshop; IJ, ImageJ; RMT, Rectangular Marquee Tool; FHT, Free Hand Tool.)

The Bland-Altman plots in Fig. 4 show the agreement between the grayscale measurements for both examiners using each ROI selection method during image analysis in Photoshop or ImageJ. The observed limits of agreement were wider for both the RMT and FHT ROI selection method when using Photoshop in comparison to ImageJ. Considering the data from both examiners, the mean difference between the echogenicity estimates obtained with the RMT method in Photoshop versus ImageJ was .44 grayscale levels (95% CI [.06–.82]; *p* < .03). In comparison, the mean difference between the echogenicity estimates obtained with the FHT method in Photoshop versus ImageJ was .66 grayscale levels (95% CI [.21–1.10]; *p* < .001). Visual analysis of the Bland-Altman plots in Figs. 3 and 4 reveal that ≤ 2 measurements were outside the limits of agreement, and no systematic errors were observed. In addition, the matrix scatter plots (Fig. 5) depict uniform coefficients of determination (*R*^2^ = .96 to .99, *p* < .001) that indicate strong positive associations among the grayscale histogram analysis measurement conditions independent of the ROI selection methods and imaging platform.

## Discussion

The assessment of muscle quality is an emergent aspect of sarcopenia screening and staging, and has important implications for geriatric medicine concerning the maintenance of musculoskeletal health. The parallel clinical management approach of two geriatric syndromes, sarcopenia and osteoporosis, are instructive in understanding the growing importance of tissue quality to clinical outcomes. Both of these age-related conditions have been associated with a diagnosis or staging process involving the use of young adult reference data obtained via dual-energy x-ray absorptiometry (DXA). DXA examinations are used to provide clinical estimates of bone mineral density (BMD) and lean body mass (LBM). However, just as bone density fails to adequately predict the pathological fracture rate of long bones (Licata, 2009), growing evidence suggests that low muscle mass is not consistently associated with poor muscle performance or adverse clinical outcomes in older adults (Cawthon et al., 2009; Hairi et al., 2010; Ismail et al., 2015). Bone quality is a broad construct associated with fracture resistance, and includes additional elements beyond BMD such as collagen quality, osteoclastic activity, and bone microarchitecture (Singer & Eyre, 2008; Licata, 2009). While it is recognized that antiosteopororic therapy results in bones that become stronger before the detection of increased BMD, there is no accepted standard for the clinical assessment of bone quality. In a similar fashion, there is no universally accepted assessment of muscle quality. Muscle quality has been expressed in formal terms by calculating specific force from the physiological cross-sectional area of a given muscle (Lieber, 2010; Krivickas et al., 2011), measuring muscle performance efficiency based on whole muscle force production relative to body size or regional LBM estimates (Cawthon et al., 2009; Scott et al., 2010), and obtaining estimates of muscle tissue composition via clinical imaging techniques (Sipilä & Suominen, 1993; Goodpaster et al., 2000; Fukumoto et al., 2012). Given the well-known age-related changes in supraspinal factors that affect net force production (Clark & Fielding, 2012), and the limitations associated with measuring specific force in clinical settings, the assessment of muscle tissue composition in older adults is garnering greater attention as a viable approach to characterize muscle quality (Marcus, Addison & LaStayo, 2013; Harris-Love et al., 2014b; Hamaguchi et al., 2014).

Indeed, quantifying muscle quality based on tissue composition may provide insights about a wide array of conditions associated with increasing age. Diminished muscle tissue composition, as measured by CT imaging at the mid-thigh and expressed as mean Hounsfield units (HU), may be associated with critical musculoskeletal health outcomes such as hip fracture incidence. An increased relative risk (RR) of hip fracture (RR/SD = 1.58, 95% CI [1.10–1.99]) was observed in a study involving 2941 adults over 70 years of age from the Health, Aging and Body Composition (Health ABC) cohort. These observations were obtained over a 6.6 year period and included model adjustments for age, race, sex, BMI, and percentage body fat. Further adjustments to this model for BMD, muscle strength, mid-thigh cross-sectional area, and functional performance status yielded a similar hip fracture risk profile (Lang et al., 2010). Additional findings from the Health ABC Study pertain to the prospective impact of muscle quality on the incidence of mobility limitations. The study cohort involved 3075 community-dwelling older adults observed over a period of 2.5 years. While the Cox’s proportional hazard ratios (HR) were 1.90 (95% CI [1.27–2.84]) in men and 1.68 (95% CI [1.23–2.31]) in women based on the upper and lower quartile of muscle cross-sectional area, meaningful values were also obtained for knee extensor strength (HR = 2.02, 95% CI [1.39–2.94] and HR = 1.91, 95% CI [1.41–2.58]), and for muscle attenuation during CT imaging (HR = 1.91, 95% CI [1.31–2.83] and HR = 1.68, 95% CI [1.20–2.35]) for men and women, respectively. However, only muscle quality (based on muscle attenuation expressed as HU at the mid-thigh) and muscle strength predicted incident mobility limitations (*p* < .05) during the construction of a single model (Visser et al., 2005).

The implementation of research findings concerning the assessment of muscle quality is dependent on identifying an accessible modality with a minimal testing burden. The clinical assessment of muscle quality using quantitative ultrasound has been explored, perhaps the most extensively, in the neuromuscular diseases (Pillen & Van Alfen, 2011). Sonographic methods have been used to aid the diagnosis of individuals with suspected neuromuscular disease. In a prospective study involving 150 symptomatic children, echogenicity derived from 4 scanning sites was used to effectively discriminate between those with and without the diagnosis of neuromuscular disease (Pillen et al., 2007). In addition, the use of quantitative ultrasound has demonstrated responsiveness in monitoring the progression of muscle pathology. Increased echogenicity corresponds with the advancement of dystrophic muscle changes in boys with Duchenne muscular dystrophy over a follow up period spanning approximately 2 years (median follow-up = 27.5 months, interquartile range = 22.5–32.0 months). Moreover, these increases in echogenicity are associated with decrements in muscle strength, ambulatory performance, and functional status in this patient population (Jansen et al., 2012). Inclusion body myositis, the most common form of intrinsic muscle disease in older adults, has been distinguished from other forms of idiopathic myopathy based on echogenicity measured at the triceps surae (Nodera et al., 2015). In contrast to the ongoing work involving neuromuscular disease, the assessment of sarcopenia has traditionally featured measures of muscle mass rather than muscle quality. The European Working Group on Sarcopenia in Older People (EWGSOP) has defined the diagnostic criteria for sarcopenia as low muscle mass with either low muscle strength or low physical performance (Cruz-Jentoft et al., 2010). However, Cawthon and associates (2015) have found limitations in predicting clinical outcomes in older men using an approach to the sarcopenia diagnosis that is based on muscle mass cut off values. They reviewed the medical records of 5,934 community-dwelling older adults participating in the Osteoporotic Fractures in Men Study to determine the association between the sarcopenia diagnosis and key clinical outcomes. Their findings indicated that a sarcopenia diagnosis based on the criteria established by the EWGSOP and others did not improve upon the use of patient age as a predictor of reported falls, mobility limitations, or mortality. Other investigators have also noted that measures of muscle strength supersede muscle mass as a significant predictor of functional deficits and disability in large cohort studies (Newman et al., 2006; Hairi et al., 2010). However, strength loss is a non-specific sign affected by multiple body systems, and pathologic muscle impairments may have a myogenic or neurogenic origin. Consequently, the assessment of muscle quality via estimates of tissue composition may have utility in the screening and diagnosis of sarcopenia, as well as the differential diagnosis process associated with neuromuscular diseases (Pillen & Van Alfen, 2011; Harris-Love et al., 2014a). The EWGSOP has proposed a staging system for sarcopenia where the loss of muscle mass alone is considered “presarcopenia”, low muscle mass compounded with either decreased strength or function is “sarcopenia”, and low muscle mass with both poor strength and function is deemed “severe sarcopenia.” Recent evidence suggests that sonographic estimates of muscle quality based on echogenicity are significantly associated with muscle strength and functional status in older adults (Fukumoto et al., 2012; Watanabe et al., 2013; Ismail et al., 2015). Thus, the exploration of diminished muscle quality as an independent criterion for presarcopenia and as an element of the sarcopenia staging process by the EWGSOP and other consensus groups may warrant further study.

In this report, two commonly used software programs (Photoshop and ImageJ) were examined to determine the reliability and measurement error associated with assessing echogenicity via grayscale histogram analysis. Moreover, the influence of the ROI selection methods on measurement reliability and agreement was also investigated. The results indicate that the two novice examiners demonstrated excellent intrarater and interrater reliability in the measurement of rectus femoris echogenicity via grayscale histogram analysis. The lower bound 95% CI of the ICC values all exceeded .97 (*p* < .001) using both the RMT and FHT selection method for the ROIs in both image analysis platforms. Overall, the limits of agreement were better for the RMT method in comparison to the FHT method, and smaller measurement errors were observed with echogenicity estimates calculated with ImageJ. Nevertheless, the statistically significant differences between the image analysis platforms and the ROI selection methods used in this study represent small absolute and proportional error estimates. The estimated measurement errors appear to be suitable for clinical and research use as the SEM values were between .72 and 1.05 grayscale levels for images with mean grayscale values that ranged from 26.55 ± 11.38 to 28.19 ± 11.57. These observed interrater measurement errors are approximately 2.6% to 4.0% of the mean grayscale values obtained from the study sample. To place these values into proper context, it may be useful to consider how estimates of muscle quality based on muscle echogenicity may distinguish between adults with relatively high and low muscle performance. Our recent findings suggest that women with high indices of grip strength in proportion to body weight exhibit low levels of muscle echogenicity as estimated with grayscale histogram analysis. Stronger women in the sample had grayscale values of 38.0 ± 17.0, whereas weaker women had grayscale values of 58.5 ± 21.0 (Ismail et al., 2015). The preliminary results indicate that the group differences in the mean grayscale values, which may distinguish between high and low muscle performance in older adults, far exceed the measurement errors using Photoshop and ImageJ cited in this study.

In addition, the matrix scatter plot and the associated *R*^2^ values show a high degree of association across ROI selection methods and the image analysis platforms. The strong association between the ROI selection methods is likely to be influenced by the longitudinal scanning images of the rectus femoris used in the analyses (Fig. 2). Both ROI selection methods have relative advantages, as the RMT method may confer benefits regarding the time needed to determine the tissue boundaries included in the analysis, and the FHT method may provide accuracy of the selected ROI based on the fascial borders of the targeted muscle. The square or rectangular appearance of the longitudinal rectus femoris image provided uniform fascial borders and a field of view for ROI selections that were readily captured using both the FHT and RMT methods. This image uniformity was reflected in the similar mean values and variance estimates of the ROI selection methods. However, the advantage of using the FHT method may be more apparent in clinical circumstances that require the ROI selection of tissue with complex anatomical borders.

Other investigators have reported good reliability for post-image acquisition grayscale histogram analysis (Pillen et al., 2003; Zaidman et al., 2010; Zaidman, Holland & Hughes, 2012; Shklyar et al., 2015; Sarwal et al., 2015; Caresio et al., 2015). Nevertheless, important differences in the assessment methodology found in the literature merit additional consideration. Echogenicity values may be affected by external factors such as the angle of the transducer with respect to the skin surface and underlying muscle bundles, the scanned image orientation (e.g., longitudinal or transverse), and the configuration of the ROI (Zaidman et al., 2008; Harris-Love et al., 2014a). Similar to this study, Caresio and associates (2015) examined the reliability of grayscale histogram analysis at the rectus femoris. Their previous work involved 20 healthy young adults and featured a maximum ROI selection method that parallels the FHT method featured in this study, along with other ROI permutations including a smaller rectangular ROI. The investigators attained acceptable reliability using their method as their ICC values were .78–.86, with a coefficient of variation (CV) ranging from 7.9% to 10.7%. The modest differences in the reliability coefficients of Caresio et al. and the findings reported in this study may be attributed to their use of transverse view images in contrast to the longitudinal view images used in the present work. Zaidman and colleagues (2008), who conducted a study involving the ultrasound imaging of the elbow flexors in 20 healthy adults, suggested that measurements involving longitudinal view images may be more reliable than the use of transverse view images. Using their technique to relate grayscale levels to backscatter via the calculation of calibrated muscle backscatter values (cMBs expressed as GSL/dB), Zaidman et al. (2008) reported that the ICCs for the cMB analysis were .93–.95 for the longitudinal view images and .73–.82 for the transverse view images.

Sarwal and colleagues (2015) also used transverse images in their ultrasound examinations involving critically ill patients admitted to the intensive care unit (ICU). The ultrasound examinations completed by these investigators included the image capture from 20 rectus femoris scans and featured ImageJ in the post-image acquisition analysis. Their ROI selection protocol was used by 4 examiners (2 trained novices and 2 experienced team members) and included the “trace method” (similar to the FHT method in the current study), as well as the “square technique” to capture a representative area of tissue echogenicity. Although the novice examiners exhibited interrater reliability ICCs of .844 (95% CI [.537–.943]), the more experienced examiners attained excellent lower bound reliability coefficient estimates values (ICC = .973; 95% CI [.935–.989]). While ICCs in isolation cannot be used to convey absolute reliability, the novice examiner ICC values in the Sarwal et al. (2015) study approach the degree of relative reliability reported by Caresio et al. (2015) for the rectus femoris—both groups used the transverse view of the target muscle group which is more difficult to capture using conventional ROI selection methods. Other investigators have obtained grayscale or cMB values from longitudinal view scans with a high degree of reliability (Zaidman, Holland & Hughes, 2012; Ismail et al., 2015). Nonetheless, the experienced examiners featured by Sarwal et al. (2015) were able to consistently assess the echogenicity of the rectus femoris in the more complex orientation afforded by the transverse view, which does attest to the value of examiner capability and training.

Importantly, Caresio et al. (2015) have carefully documented that variation in ROI size significantly impacts echogenicity levels expressed as grayscale values. Some investigators have noted that large ROIs may adversely affect echogenicity estimates because of the hyperechoic nature of intercompartmental fascia in muscle groups with bipennate or multipennate architecture. However, it has been noted that non-pathologic muscle tissue echogenicity varies among differing muscle groups and that this variation is likely a function of muscle ultrastructure, fiber orientation, and relative amounts of non-contractile tissue (Pillen & Van Alfen, 2011; Caresio et al., 2015). Consequently, ROI selection methods that incorporate full muscle thickness dimensions may appropriately characterize the normal anatomical variation among muscle groups, and account for the inherent heterogeneity of muscle tissue echogenicity (Zaidman et al., 2008). The call to incorporate superficial geometric ROI selection methods into cMB and grayscale histogram analyses has emanated from investigators involved in the diagnosis and management of neuromuscular disorders (Zaidman, Holland & Hughes, 2012; Shklyar et al., 2015). Recent findings suggest that quantitative backscatter analysis and grayscale values from superficial ROIs within the tibialis anterior were negatively associated with performance scores obtained using a standardized functional assessment battery (i.e., the North Star Ambulatory Assessment) in people with Duchenne muscular dystrophy (Shklyar et al., 2015). In contrast, quantitative backscatter analysis and grayscale values from larger ROIs involving greater scanning depth were not associated with the functional performance scores of the participants. Moreover, it has been noted that the use of fixed height superficial ROIs may be advantageous for establishing the cMB estimates of echogenicity between two different ultrasound machines by limiting measurement variability (Zaidman, Holland & Hughes, 2012). However, the observation that excessive ultrasound beam attenuation occurs in muscles with substantial dystrophic changes may not fully apply to the study of age-related muscle dysfunction (Heckmatt et al., 1989; Jansen et al., 2012). Watanabe et al. (2013) examined the echogenicity of the rectus femoris using Photoshop in 184 community dwelling older men (aged 65–91 years). Their findings suggest that peak isometric knee extension torque is significantly associated with the grayscale levels obtained with a full muscle thickness ROI using the FHT method (*r* = − 0.33, *p* < 0.001). In a similar vein, a full muscle thickness ROI using the FHT method at the rectus femoris for grayscale histogram analysis was significantly associated with grip strength scaled to body weight in a sample of community dwelling adult women (Ismail et al., 2015). The multiple regression model with age and echogenicity as predictor variables yielded a *R*^2^ value of .53 (*p* = .001), and the partial correlations suggested that grayscale levels had a larger degree of association with strength in comparison with age (*r*_*xy*⋅*z*_ = − .52 versus *r*_*xy*⋅*z*_ = − .38). While the fixed height superficial ROI method has been effectively taught to examiners involved in the study of neuromuscular disorders (Shklyar et al., 2015), the full muscle thickness approach to ROI selection has also been shown to be very reliable in studies involving samples with older adults (ICC = .96 and CV = 4.2%) (Watanabe et al., 2013).

While the RMT and FHT methods for obtaining full muscle thickness ROI selections appear to be reliable, direct comparisons with alternative approaches such as the fixed height superficial ROI method are beyond the scope of the study objectives. Additional research may be needed to understand if ultrasound beam attenuation within the far-field view of larger ROIs negatively affects the previously reported relationship between muscle echogenicity estimates and muscle performance in older adults. This study was also limited by the investigation of quantitative ultrasound in only the rectus femoris. Although the rectus femoris is an important muscle group in the sonographic assessment of sarcopenia and other chronic disorders (Watanabe et al., 2013; Ismail et al., 2015; Hernandez et al., 2015), selected upper extremity muscle groups may merit further study as ultrasound-assisted sarcopenia screening and staging is further developed to accommodate non-ambulatory populations (Harris-Love et al., 2014b). This report addresses sources of error related to the post-image acquisition analysis by the examiners, the image analysis software used, and the inherent variation of the scanned images from the sample of older adult participants. However, the sources of error associated with image capture, and the variability of echogenicity levels secondary to different ultrasound machines and transducers were not explored in this study. Finally, while more current versions of Photoshop are available in comparison to the software program featured in this work, the reported ROI selection strategies and echogenicity estimate methods for the musculoskeletal images are similar to other contemporary research reports (Shklyar et al., 2015; Caresio et al., 2015).

## Conclusions

In summary, the Rectangular Marquee Tool and the Free Hand Tool region of interest selection methods yielded high intra and interrater reliability among two novice examiners measuring muscle echogenicity via grayscale histogram analysis. The minimal measurement error exhibited by the examiners demonstrates that the region of interest selection methods used with Photoshop and ImageJ are suitable for the post-acquisition image analysis of tissue echogenicity in older adults. The comparable grayscale levels measured with the two region of interest selection methods may have been aided by the geometric configuration of the rectus femoris images boundaries in the longitudinal scanning view. Although the Rectangular Marquee Tool facilitates a rapid selection of the region of interest, the Free Hand Tool may be an advantageous method for muscles or tissues with irregular boundaries. Finally, while smaller measurement errors were observed with echogenicity estimates calculated with ImageJ in comparison to Photoshop, these differences were minimal and both image analysis software programs generated similar results.

## Supplemental Information

10.7717/peerj.1721/supp-1 Supplemental Information 1 Flowchart of the study

## Additional Information and Declarations

### Data Availability

The following information was supplied regarding data availability: The data is owned by the US Department of Veterans Affairs and is therefore restricted.
