# Supplementary material for: Ultrasound estimates of muscle quality in older adults: reliability and comparison of Photoshop and ImageJ for the grayscale analysis of muscle echogenicity
Source: PeerJ. 2016 Feb 22;4:e1721. doi: 10.7717/peerj.1721 (PMC4768702; doi:10.7717/peerj.1721)
Supplement: Supplemental Information 1 [file peerj-04-1721-s001.docx]

CONSORT diagram: Observational study

Assessed for eligibility

(n = 18)

# Enrollment

Randomized (n = 18)

Excluded (n = 0)

Not meeting inclusion criteria

(n = 0)

Refused to participate

(n = 0)

#

**Follow up**

Analyzed (n = 18)

Excluded from analysis

(n =0)

Analyzed (n = 18)

Excluded from analysis

(n =0)

# Analysis

Lost to follow up:

n/a

Lost to follow up:

n/a

Allocated to

**Examiner 2**

(n = 18)

No intervention; image analysis order randomized for each examiner

Allocation

Allocated to

**Examiner 1**

(n = 18)

No intervention; image analysis order randomized for each examiner
